# Hydrodissection using 10% dextrose before focal therapy of prostate cancer: Initial experience

**DOI:** 10.1002/bco2.70073

**Published:** 2025-09-02

**Authors:** Julien Anract, Marie Florin, Laura Larnaudie, Michael Peyromaure, Nicolas Barry Delongchamps

**Affiliations:** ^1^ Cochin Hospital, Urology Department APHP, Université Paris Cité Paris France; ^2^ INSERM U1151, Team 5 Institut Necker Enfants Malades (INEM) Paris France; ^3^ Hôpital Tenon, Radiology Department APHP, Sorbonne Université Paris France; ^4^ Cochin Hospital, Pathology Department APHP, Université Paris Cité Paris France

**Keywords:** dextrose, Focal therapy, hydrodissection, periprostatic nerve block, prostate cancer

## Abstract

**Objectives:**

To evaluate the feasibility and safety of hydrodissection of the prostato‐rectal space using 10% dextrose for focal therapy of posterior prostate tumours.

**Patients and methods:**

We included consecutive patients who underwent focal therapy for a posterior prostate tumour with a prior injection of 10% dextrose in the prostato‐rectal space, between October 2024 and February 2025. The main outcomes were to evaluate the space created using this technique. As the technique used for hydrodissection was modelled on periprostatic nerve block, we analysed a cohort of patients who underwent transperineal prostate biopsies with periprostatic nerve block using 20 ml of lidocaine, to compare the prostato‐rectal spaces created by 10% dextrose and by lidocaine.

**Results:**

A total of 11 patients underwent a focal therapy with a prior 20 ml 10% dextrose hydrodissection of the prostato‐rectal space. Fifteen patients who underwent prostatic biopsies using a periprostatic nerve block (20 ml of lidocaine), with similar characteristics, were included. The median prostato‐rectal space created with dextrose and with lidocaine was 8.9 [8.0; 9.9] and 6.7 [6.4; 8.4] mm, respectively (p = 0,17). The prostato‐rectal space decreased slower with dextrose: 0.03 mm/min vs 0.1 mm/min (p = 0,02). The prostato‐rectal space was higher at the end of focal therapy procedures (7.9 vs 6.6 mm, p = 0,033), despite a longer procedure time in focal therapy (37 vs 8 min, p < 0,001). At the end of focal therapy procedures, all patients had a prostato‐rectal space > 5 mm. No hydrodissection‐related adverse event was observed.

**Conclusions:**

These initial results suggest that hydrodissection of the prostate–rectal space using 20 ml 10% dextrose, injected following a standard periprostatic nerve block protocol, is feasible, reproducible and safe for a focal therapy procedure for localized posterior prostate tumours.

**Abbreviations**
MRI: magnetic resonance imaging
PR: prostato‐rectal
US: ultrasound

## 1 INTRODUCTION

The management of prostate cancer continues to evolve rapidly, with substantial advances being made in gland‐sparing treatments, so‐called focal therapy. Multiple strategies are currently being evaluated. Notably, the recent spreading of transperineal prostatic biopsies paved the way for the development of transperineal needle‐based approaches, using different energies.^1^

Whatever strategy is proposed, a patient proper selection needs to be carefully addressed to allow optimal efficacy and safety: efficacy is probably optimized when patients harbour small ISUP 2 tumours, without secondary significant disease,^2^ although lack of oncological results does not allow proposing clear recommendations.^3^ Safety is optimized when tumours to be ablated are not located in close proximity to critical anatomical structures such as the rectum, vascular bundles or urinary sphincter.

The majority of prostate cancers are located in the peripheral zone.^4^ Hence, the proximity of the index tumour to the rectum is probably one of the most common reasons for excluding a patient from a focal therapy strategy. In these patients, the risk is either to induce a rectal fistula,^5^ or to undertreat the tumour, leading to immediate or early treatment failure.

Ultrasound‐guided hydrodissection is a standard technique that serves as an effective adjuvant to separate adjacent tissues in different clinical situations, essentially to preserve adjacent anatomical structures from thermal damage.^6^ It has been evaluated in different organs,^7^, ^8^ using saline^9^ or dextrose solution^10^. In patients with prostate cancer, hydrodissection of the prostato‐rectal (PR) space has demonstrated its value in mitigating adverse events during focal therapy and external beam radiation therapy.^11^ In this indication, polyethylene‐glycol‐based or hyaluronic‐acid‐based hydrogels were developed to maintain the PR space for several weeks.^12^, ^13^ However, a recent study evaluated the feasibility of a hydrogel spacer in the context of cryotherapy for prostate cancer.^14^

The thickness and duration (i.e. resorption rate) of PR space created by hydrodissection do not have the same relevancy and importance depending on the treatment setting. In the case of focal therapy, maintaining the PR space stable for weeks does not appear mandatory. However, it has to be stable enough to protect the rectum from thermal injury during the entire procedure. To the best of our knowledge, no study has evaluated the feasibility of hydrodissection using dextrose solution in patients undergoing focal therapy for prostate cancer.

We report here our experience in hydrodissection using dextrose solution in patients undergoing focal therapy for posterior prostate tumours. As the protocol of hydrodissection was modelled on our observations during periprostatic nerve block for prostatic biopsies (using 20 ml of lidocaine), we aimed to compare the PR space created using 20 ml dextrose with the PR space (focal therapy procedure) created using 20 ml of lidocaine (biopsy procedure).

## 2 PATIENTS AND METHODS

### 2.1 Data collection

We retrospectively reviewed the medical files and intra‐operative imaging data of i) patients who underwent focal therapy for a posterior prostatic tumour with prior hydrodissection using 10% dextrose injection, and ii) patients who underwent transperineal prostatic biopsies under local anaesthesia with peri‐prostatic nerve block in our academic centre between October 2024 and February 2025.

We excluded from the analysis patients who had not given their consent to participate in a retrospective analysis, had undergone any previous treatment for prostate cancer, or for whom the intra‐operative imaging data were not available for review.

### 2.2 Interventions

Transperineal targeted prostatic biopsies were guided by magnetic resonance imaging/ultrasound (MRI/US) fusion, under local anaesthesia. Local anaesthesia was a US‐guided periprostatic nerve block, according to the following protocol: positioning of the US probe on the right prostate and seminal vesicle in a parasagittal median view. Insertion of a 20 G needle in the space between the rectum and the prostate base, injection of 10 cc of 1% Lidocaine from the base to the apex (needle is inserted to the prostate base, and the liquid is injected during the extraction of the needle, to avoid dissection of the rectal mucosa). The same procedure was performed on the left side of the prostate, for a total of 20 cc injected in the space.

**FIGURE 1:**
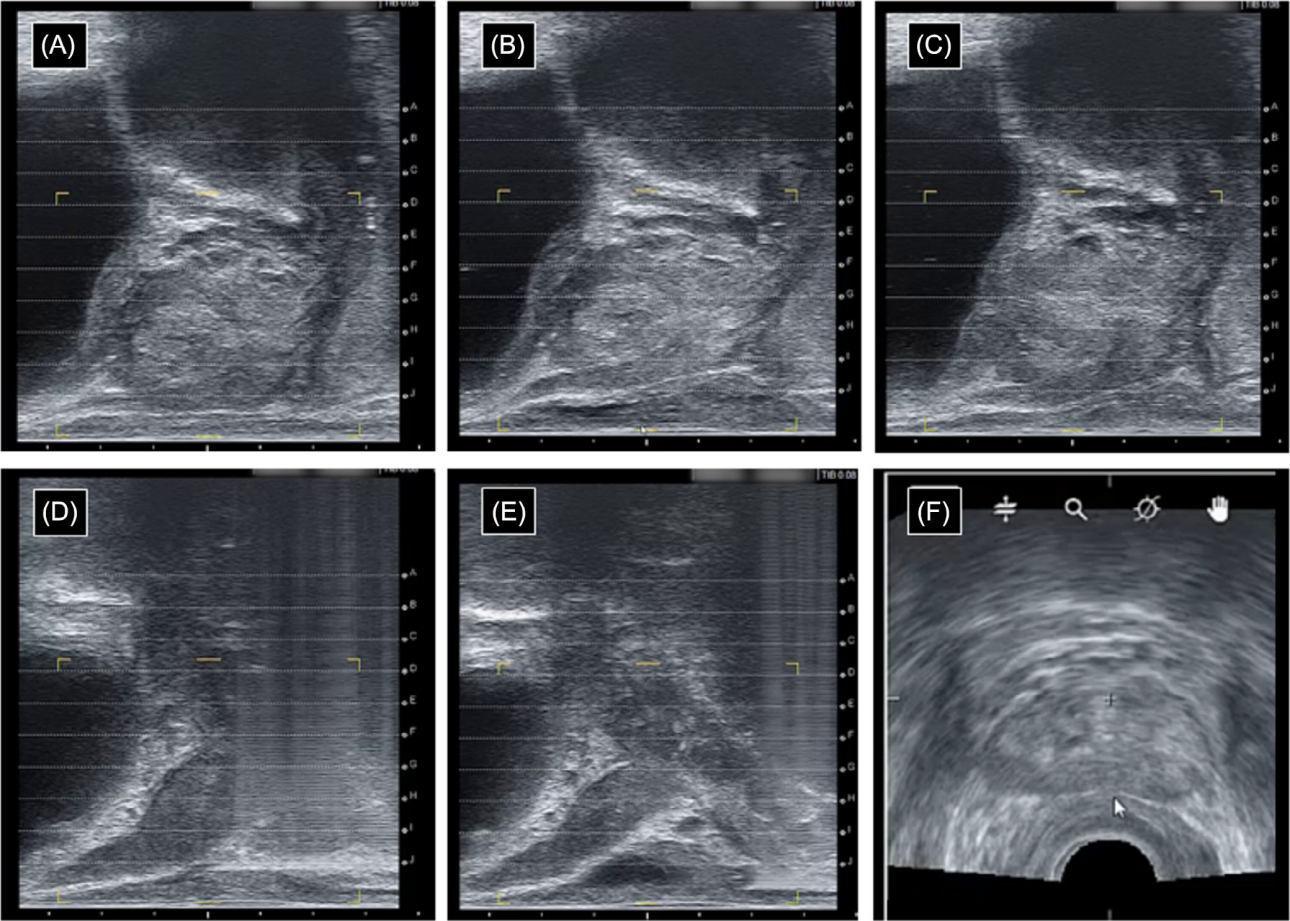
Ultrasound images of the hydrodissection technique used during focal therapy. (A) Sagittal median view of the prostate before the hydrodissection (T0). (B) Sagittal median view of the prostate immediately after the hydrodissection (T1). (C) Sagittal median view of the prostate at the end of the procedure (T2). (D) Sagittal para‐median view of the prostate during the insertion of the hydrodissection needle in the PR space. (E) Sagittal para‐median view of the prostate during the extraction of the hydrodissection needle from the PR space. (F) Axial view of the prostate immediately after hydrodissection (T1), the white narrow indicating the posterior surface of the prostate.

Hydrodissection prior to focal therapy was performed with 10% dextrose solution, using a protocol modelled on periprostatic nerve block: the US probe was positioned on the right in a similar parasagittal median view, and a similar 20 G needle was introduced in the space between the rectum and the prostate base (Figure 1D). We then injected 10 cc of 10% dextrose from the base to the apex (Figure 1E). The procedure was repeated on the left side of the prostate with another 10 cc of 10% dextrose. Symmetry of the space created was verified on an axial reconstruction (Figure 1F).

This study was approved by the local ethics committee board: acceptation number: AAA‐2024‐10 050.

### 2.3 Measurements

Measurements were performed on the recorded images using the TRINITY software (Koelis®, France). All measures were performed on a sagittal median US view of the prostate. Measures of PR space were evaluated at different times and defined as the shorter distance between the posterior capsule of the prostate mid‐gland and the anterior rectal wall. Baseline PR space was measured before any hydrodissection (time T0, Figure 1A). The PR space created was measured immediately after hydrodissection (time T1, Figure 1B). The remaining PR space was measured at the end of the procedure (prostate biopsy or focal therapy) (time T2, Figure 1C). All procedures were precisely timed using the recorded images. The procedure duration was defined as the time between the end of the periprostatic nerve block or hydrodissection and the removal of the US probe at the end of the procedure. Assuming that the decrease of the space after hydrodissection was linear, we calculated for each patient the resorption rate using the following formula: PR space at T1 – PR space at T2/T2–T1.

### 2.4 Statistical analysis

Continuous variables are expressed as medians and quartiles m [q1; q3]. Measures of PR spaces at T0, T1 and T2 were compared using one‐way ANOVA, with paired analysis of data. Multiple comparisons between different times were conducted only if one‐way ANOVA test was statistically significant. PR spaces were compared between the biopsy and focal therapy groups using an independent unpaired two‐tailed T test. All the analysis was conducted using GraphPad Prism® v.10.4.1, using a p value <0.05 as statistically significant.

## 3 RESULTS

Fifteen patients were included in the biopsy cohort, and 11 patients in the focal therapy cohort. The two cohorts had comparable characteristics (Table 1). The median [q1;q3] duration of procedures, corresponding to the time length between T1 and T2, was 8 [7; 9] min and 37 [30; 40] min for biopsy and focal therapy, respectively.

**TABLE 1:** Patients' characteristics.

	PB, n = 15	FT, n = 11	*p Value*
Age	68 [64; 72]	74 [65; 77]	*0.31*
PSA (ng/ml)	10.1 [4.4; 8.4]	6.6 [3.5; 7.0]	*0.49*
Prostate Volume (cc)	48 [37; 77]	42 [32; 46]	*0.12*
Target Largest Axis (mm)	10 [7; 13]	11 [9; 13]	*0.74*
Distance Tumour – Rectum (mm)	3 [1; 8]	3 [2; 5]	*0.37*

In the focal therapy cohort, 7 and 4 patients were treated using targeted microwave ablation and irreversible electroporation, respectively. The fusion system, US probe and hydrodissection protocol were identical for every case. The median [q1;q3] distance between the targeted tumour and the anterior rectal wall was 3 [2; 5] mm (measured on axial T2 weighted MRI, as the shorter distance between the posterior tumour surface and the anterior rectal wall).

**FIGURE 2:**
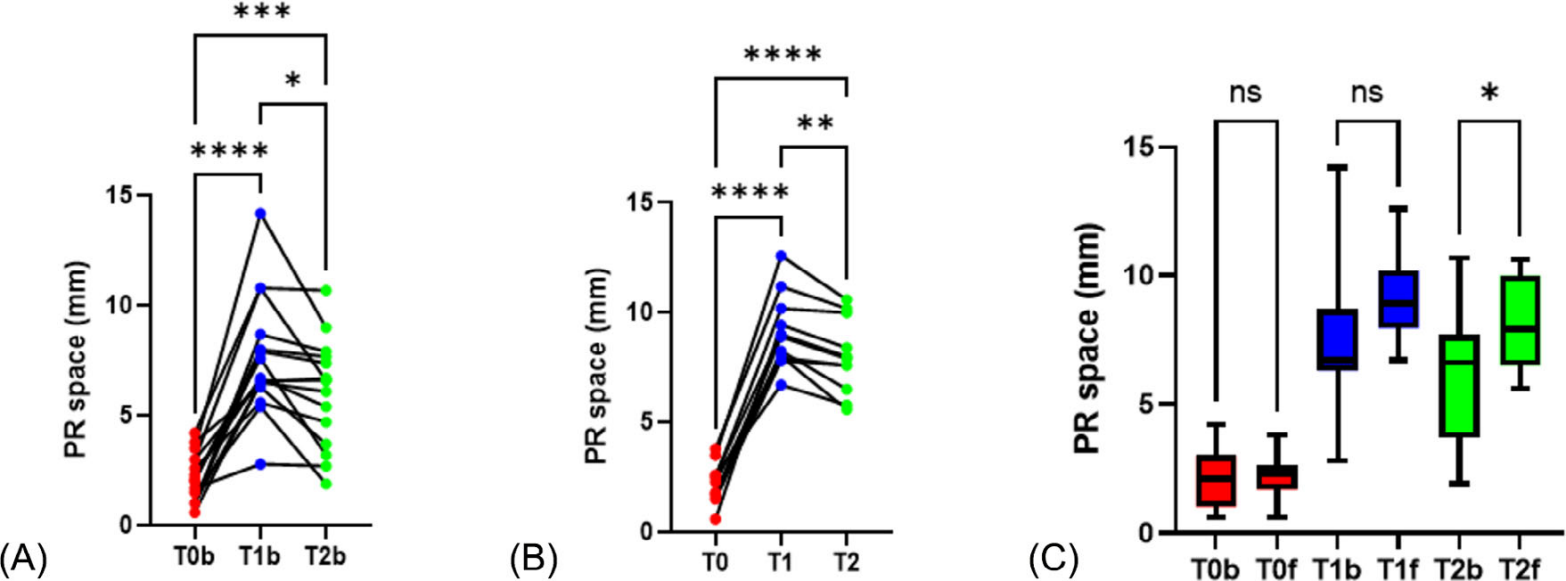
Distribution of PR spaces measured during biopsy and focal therapy procedures at time T0, T1 and T2. (A) Comparison of median PR space measured at T0, T1 and T2 with local anaesthesia during biopsy procedures. One‐way ANOVA, paired analysis. R squared = 0.765, p < 0.0001 / Multiple comparison: T0 vs T1 p < 0.0001; T0 vs T2 p < 0.0001; T1 vs T2 p = 0.0015. (B) Comparison of median PR space measured at T0, T1 and T2 with 20 cc of 10% dextrose solution during focal therapy procedures. One‐way ANOVA, paired analysis. R squared = 0.984, p < 0.0001 / Multiple comparison: T0 vs T1 p < 0.0001; T0 vs T2 p < 0.0001; T1 vs T2 p = 0.0015. (C) Comparison of PR space measured during biopsy (T0b, T1b and T2b) and focal therapy procedures (T0f, T1f and T2f). One‐way ANOVA, paired analysis. R squared = 0.673, p < 0.0001/Multiple comparison: T0b vs T0f p > 0.99; T1b vs T1f p = 0.17; T2b vs T2f p = 0.0337.

During biopsy procedures, the median PR space was 2.1 [1.3; 3.0], 6.7 [6.4; 8.4] and 6.6 [4.2; 7.5] mm, before hydrodissection (T0), immediately after hydrodissection (T1) and at the end of the procedure (T2), respectively (p < 0.0001) (Figure 2A). Between T0 and T1, the PR space median increase was 4.6 mm (p < 0.0001). Between T1 and T2 (median delay 8 minutes), the PR space median decrease was 0.7 mm (p = 0.0015) (Figure 2A).

During focal therapy procedures, the median PR space was 2.3 [1.7; 2.6], 8.9 [8.0; 9.9] and 7.9 [7.1; 9.2] mm at T0, T1 and T2, respectively (p < 0.0001) (Figure 2B). Between T0 and T1, the PR space median increase was 6.6 mm (p < 0.0001). Between T1 and T2 (median delay 37 minutes), the PR space median decrease was 1 mm (p = 0.0015).

During focal therapy procedures, hydrodissection allowed a satisfying PR space, and no further injection of 10% dextrose was performed. No adverse event related to hydrodissection or rectal injury was reported.

Comparing the PR spaces from the biopsy and focal therapy cohorts, there was no difference at T0 (p > 0.99) and T1 (p = 0.17). The PR space at T2 was significantly higher in the focal therapy cohort compared to the biopsy cohort: +1.3 mm – 95% CI = [0.12–3.9; p = 0.033] (Figure 2C).

The median resorption rate of the PR space was 0.1 (0.02;0.52) mm/min and 0.03 (0.015;0.036) mm/min for the biopsy cohort and focal therapy cohort, respectively (p = 0.02).

## 4 DISCUSSION

Rectal complications, especially uro‐rectal fistula, are serious and unfortunately common complications of prostate cancer treatments.^15^ Focal therapy, initially intended to reduce treatment‐related morbidity, may still cause significant damage when targeting tumours located too close to the rectal wall, regardless of the energy source used.

Targeted microwave ablation and irreversible electroporation are two novel needle‐based focal therapy modalities for localized prostate cancer.^16^, ^17^, ^18^ In our setting, both techniques are performed under MRI/US guidance using tridimensional US and organ‐based tracking fusion technology (Koelis® Trinity), allowing for optimal precision of ablation. During microwave procedures, multiple and consecutive thermal ablations are applied using the same needle.^18^ In contrast, electroporation is performed with four needles inserted together transperineally through a grid to create the ablation zone.^19^ While temperature is reported to be between 70 and 90°C in the microwave ablation zone, some authors suggested that electroporation was “athermic” a statement that is largely debated. In a systematic review of 14 studies and nearly 900 patients treated with electroporation, Prabhakar et al.^20^ noted a 0.2% rate of rectourethral fistula, confirming this complication to be rare. In the PRESERVE study, a prospective, non‐randomized, pivotal trial of subjects in the United States from 17 clinical centres, one patient out of 121 developed a delayed rectoprostatic urethra fistula, which presented at 3 months post‐treatment. The patient had an 18 mm lesion located in the posterior peripheral zone. Hydrodissection was not used.^21^ Microwave ablation is a much younger technique in its development, performed only since 2017. Previous reports did not mention any rectum‐related adverse events, especially rectal fistula.^18^ Nonetheless, it is important to note that a distance between the rectum and the tumour < 5 mm was an exclusion criterion.

Other needle‐based focal therapy modalities, such as cryotherapy or laser ablation, have also restrictions at the posterior capsule because of the risk of thermal damage.

HIFU, in contrast, is a non‐needle‐based modality of transrectal thermal energy delivery and has the advantage of precise tissue ablation for posteriorly located lesions.^22^ Although authors agree that focal HIFU is much more appropriate than focal cryotherapy for posterior tumours, it is not out of risk of thermal damage. Rectal fistulae rate after HIFU is between 0 and 3%.^23^ The majority of cases are reported after whole‐gland treatments or when used as a salvage treatment after brachytherapy or external beam radiation therapy. Although we may expect safer results after focal HIFU, fistulae have also been reported in some series.^24^, ^25^

From our point of view, and more importantly that the choice of the energy, the three key elements to prevent rectal damage are to optimize intra‐operative guidance, monitor temperature and stay as much as possible away from the rectal wall. Intra‐operative guidance is now possible with a range of different technologies offering software‐based fusion for focal therapy. Temperature monitoring is, however, not possible when using microwave ablation or standard electroporation. A new generation of electroporation systems (Surgnova® Technology) allows monitoring of temperature at the tip of each of the needles used, and postponing ablation at any time at the surgeon's discretion. Further studies are needed to evaluate if this technology allows higher safety rates.

The third key element concerns the distance to the rectal wall, and the optimal and safer way to achieve a security margin of at least 5 mm to treat the tumour and decrease the recurrence rate. This retrospective evaluation confirms that the PR space created with Lidocaine is transient and not adapted for a focal ablation. Although the PR space induced looks similar to that created with 10% dextrose, it decreased rapidly, and around 40% of the patients had a PR space beyond 5 mm at a time of 7 minutes after the injection. These results are similar to those reported by Rosenberg et al.^9^ They treated 10 patients with cryotherapy using saline solution for hydrodissection. However, due to continuous fluid absorption, repeated injections were necessary. Consequently, the total saline volume administered per patient ranged from 150 to 500 ml, and one uro‐rectal fistula was reported.

Hydrodissection using 10% dextrose was performed using the same protocol as periprostatic nerve block, because the technique is well known and considered feasible and safe.^26^ This technique is indeed the most commonly used local anaesthetic method for prostatic biopsies and is recommended as standard of care by the European Urological Association guidelines.^3^ The PR space created with 10% dextrose had a median value of 8.9 mm at T1 (median increase of 5.6 mm). It was not statistically superior to that obtained with Lidocaine during local anaesthesia. However, while biopsy duration was much shorter (7 min versus 37 min), it was statistically superior to the PR space created with periprostatic nerve block at the end of the procedure (T2). Interestingly, none of the patients had a distance of less than 5 mm from the rectal wall at the end of focal therapy procedures. The resorption rate of 0.03 mm/min allowed for losing less than 1 mm of PR space every 30 minutes, which makes it quite comfortable for needle procedures lasting less than one hour in most cases. More importantly, as shown in Figure 2B, the slope of the decay was homogeneous. These results therefore suggest that the technique is repeatable and reproducible, which is probably the most desired effect.

An important aspect of hydrodissection is that it should not induce imaging artefacts and decrease the reliability of image guidance. One may argue that the injection of liquid between the organ and US probe may affect the quality of the images. However, in our study, we did not experience any difficulty related to the hydrodissection liquid for US images readability. Also, US 3D image acquisition before US‐MRI fusion was performed after hydrodissection in order to prevent any fusion error.

This retrospective analysis has limitations. In addition to its retrospective design, the limited number of patients may have introduced bias. Furthermore, the PR space was assessed solely on a strict sagittal view, which may not fully capture variability in spacer diffusion — although no significant heterogeneity was observed visually. Also, we could not retrospectively evaluate if the US probe itself could modify the PR space when manipulated during surgery. Further studies with more patients are needed to confirm these initial results, especially in a prospective fashion, with a randomized comparative design.

## 5 CONCLUSIONS

Hydrodissection of the PR space using 10% dextrose seems to be feasible and safe in the setting of focal therapy for localized posterior prostate cancer. Our comparison with the PR space created during periprostatic nerve block suggests that Lidocaine may not allow a PR space stable long enough to safely perform focal therapy. Prospective studies are needed to confirm these initial results.

## CONFLICT OF INTEREST STATEMENT

Authors declare to have no conflict of interest for this publication.
